# Retraction Note: Invasion inhibition in pancreatic cancer using the oral iron chelating agent deferasirox

**DOI:** 10.1186/s12885-023-11677-6

**Published:** 2023-12-11

**Authors:** Shogo Amano, Seiji Kaino, Shuhei Shinoda, Hirofumi Harima, Toshihiko Matsumoto, Koichi Fujisawa, Taro Takami, Naoki Yamamoto, Takahiro Yamasaki, Isao Sakaida

**Affiliations:** 1 Department of Gastroenterology and Hepatology, Yamaguchi University Graduate School of Medicine, 1-1-1 Minami-Kogushi, Ube, Yamaguchi, 755-8505 Japan; 2 Department of Oncology and Laboratory Medicine, Graduate School of Medicine, Yamaguchi University, Ube, Yamaguchi, Japan

**Retraction Note: BMC Cancer 20, 681 (2020)**

**https://doi.org/10.1186/s12885-020-07167-8**

The Editor has retracted this article. After publication, concerns were raised regarding highly similar images in Fig. 2a (24 h, DFX 10 μm and DFX 100 μM), which the authors addressed by a Correction [1]. However, further concerns have been identified by the publisher, specifically:

Fig. 3c and 3i 0h and 24h control images appear to be the same.

Fig. 3c 50 and 100 uM group 0h images appear highly similar (flipped horizontally)

The authors have also found three other misused images in Fig. 3c (NSC23766 50 µM 0h) and i (ML141 20 µM and 40 µM 0h), and incorrect errors bars in the data presented in Fig. 4a.

The Editor therefore no longer has confidence in the presented data.

All authors agree to this retraction.
